# Low-Cost Heating Modalities Allow the Detection of Biomarkers for Plant Infection Using Rapid Evaporative Ionization Mass Spectrometry (REIMS) That Are Pathogen Specific

**DOI:** 10.1021/acs.analchem.5c00226

**Published:** 2025-06-27

**Authors:** Alice Flint, Ryan Weir, Luis A. J. Mur, Simon J. S. Cameron

**Affiliations:** † School of Biological Sciences, Institute for Global Food Security, 1596 Queen’s University Belfast, Belfast BT9 5DL, United Kingdom; ‡ Department of Life Sciences, 1026 Aberystwyth University, Aberystwyth SY23 3FL, United Kingdom

## Abstract

Agricultural pathogens reduce annual crop yields by up to 40% and present a barrier to improving crop production to a level by which it will be able to support a global population of 9 billion by 2050. Current diagnostic methods are slow and lack specificity and typically rely on visual signs of infection, which appear late in the infection cycle. In this work, we explored whether the ambient ionization method rapid evaporative ionization mass spectrometry (REIMS) could be used to detect pathogen-specific biomarkers of infection against two important pathogens: the nematode and bacteria in the tomato plant (). Unlike previous implementations of REIMS for human clinical diagnostics, we explored the use of low-cost heating modalities in the form of a 450 nm laser and soldering iron (both below $200). After optimization, we found that the 450 nm laser provided the highest level of diagnostic classification accuracy and, importantly, could distinguish between infection causes with pathogen-specific biomarkers. This shows both the novel utility of REIMS for analysis of plant material, which would allow *in situ* and high-throughput analysis by the community for a broad range of plant metabolome applications, and also the potential of low-cost 450 nm lasers for use beyond this.

## Graphical abstract

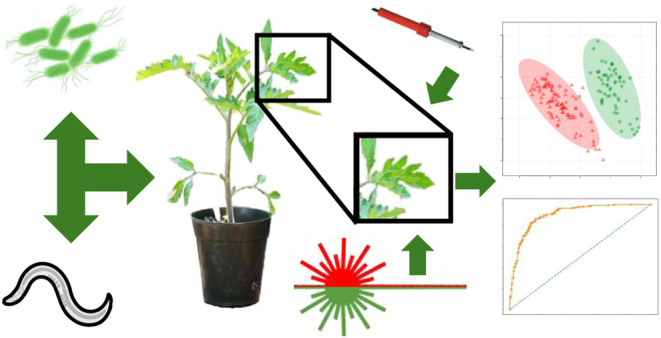

To support a predicted global population of over 9 billion people by 2050,^1^ agricultural production will need to increase markedly. One of the barriers to this is the burden of agricultural pathogens and pests, which reduce annual crop yields of some crops, such as rice, by over 40%.^2^ The use of agricultural pesticides has allowed substantial increases in productivity, but comes with associated risks of the development of resistant pathogens and environmental damage due to runoff^3^ and off-target effects.^4^ Microbial pathogens (including bacteria, fungi, parasites, and viruses) are common pathogens in agricultural crops. Due to the broad range of plant pathogens, diagnostics can be a challenge, and visible signs of infections typically appear at a point where the infection is established. Earlier detection/diagnosis of plant pathogens could, therefore, allow for earlier and more effective interventions. This could decrease the overall use of agricultural pesticides as infection would be less established and affect a smaller proportion of crops, and also prevent the spread of pathogens within an agricultural setting.^5^ Plant parasitic nematodes (PPNs) are globally distributed, soil-borne pests, which exert a significant economic burden on global agriculture.^6^ Through costs associated with control and crop yield loss, it has been estimated that these parasites are responsible for economic losses greater than £100 billion per year.^7^ Diagnosis of PPN crop infection is very challenging based on visual inspection of plant foliage. Therefore, it requires assessment of crop roots in the field and collection of PPN tissue for laboratory-based molecular diagnostics and/or analysis of soil samples for cysts. As a result, current PPN diagnostics lack throughput, convenience, and sensitivity. Bacterial plant pathogens pose additional challenges to agricultural productivity, largely linked to their faster growth rate and ease of dispersal compared to PPN. Symptoms of plant bacterial infections are typically more visually identifiable due to the impact on the above-soil plant structures, particularly leaves and fruiting bodies. Nevertheless, at this point, bacterial infections are typically well-established and will prevent early interventions to limit pathogen spread and associated impact on crop productivity.^8^

Current methods for diagnostics can be based on the observation of visual symptoms by trained personnel, but this can be costly and time-consuming and, for many bacterial pathogens, not species-specific. Molecular methods are increasingly being developed for the detection of plant pathogens, particularly the use of polymerase chain reaction (PCR) assays, loop-mediated isothermal amplification (LAMP) assays, and high-throughput sequencing. However, these are still costly and time-consuming, which prevents their broad adoption.^9^ When they are used, due to their cost, it is not typically feasible for them to be used as a screening tool. Mass spectrometry has a much lower cost-per-sample than many molecular methods, albeit with a typically higher capital expenditure required for instrumentation. Nevertheless, this offers the potential for low-cost and potentially high-throughput analysis of plant material for screening and surveillance.

Mass spectrometry-based metabolomics has been used to elucidate host–pathogen interactions during plant infections and potentially identify metabolite-based traits that could be used to breed more resistant varieties of crop plants.^10^ Fewer studies have used this approach as an alternative method of diagnosis. Success has been seen using both liquid chromatography^11^ and gas chromatography,^12^ or a combination of both separately,^13^ coupled to mass spectrometry, but has typically focused on just one pathogen. These approaches also require time-consuming and resource-intensive extractions and analytical runs, which limit their potential for screening and/or rapid diagnostics. Techniques within the field of ambient ionization mass spectrometry are characterized by an ability to perform ionization on samples in a minimally modified form in their native environment, with many taking place in air.^14^ Such approaches have the potential to make mass spectrometry analysis more accessible, less costly, and with higher-throughput than conventional methods. One such technique is rapid evaporative ionization mass spectrometry (REIMS) which uses heating to vaporize a sample and release gas-phase ions which are analyzed through a mass spectrometer.^15^ The technique was initially developed as a tool for the intraoperative identification of cancerous tissues during surgery,^16^ but saw broad application to clinical microbiology diagnostics,^17^ food authenticity analysis,^18^ and high-throughput screening,^19^ among others, due to the utilization of various modalities for sample heating, including laser ablation.^20^ These, however, have been typically costly and would likely pose a barrier to broad adoption. In this work, we explored a range of low-cost heating modalities (namely, a 450 nm laser and soldering iron), which cost approximately £150/$200 each, and optimized them for the analysis of plant leaf material. Each was then utilized in the analysis of plant leaf material from plants that had been infected with either a PPN () or bacterial () pathogen against uninfected controls, with the intention of identifying pathogen-specific biomarkers.

## Experimental Section

### Growth of Plant Material

Tomato plants ( variety “Moneymaker”) were cultivated from seedlings for this work. Seeds were planted individually in seed trays and in autoclaved compost. They were kept in a temperature-controlled room at 22 °C with automated lighting on a 16 h light and 8 h dark cycle, with regular watering. Once seedlings had grown to an appropriate size, they were transplanted into individual growth pots and kept in growth chambers. A total of eight plants were kept in each growth chamber that operated at the same temperature and light/dark cycle as before. During infection periods, experimental groups were mixed between growth chambers to minimize variation. For analysis, leaves from the same level of each plant were detached and placed onto a Columbia Blood Agar plate (E&O Laboratories, U.K.). After analysis, leaves were placed onto a Columbia Blood Agar plate (E&O Laboratories, U.K.) inside a dark chamber and on a lit platform. They were imaged using an 8MP SLR camera.

### Heating Modalities for REIMS

Three heating modalities were adapted for use in the analysis of plant leaf biomass: a 10W (1–10W in 0.5W increments) carbon dioxide laser (A.R.C., Germany) capable of operating in continuous wave and pulsatile operation (1 to 500 Hz in staggered increments), with approximate cost of £35,000, and mounted to an XYZ gantry robot (igus, U.K.), a 3W 450 ± 5 nm continuous wave laser engraver (HomdMarket, China), with approximate cost of £150, and a 70W temperature adjustable soldering iron (Weller Tools, Germany), with approximate cost of £150. All heating modalities were operated according to the manufacturer’s instructions and necessary safety considerations. The CO_2_ laser was operated through automated control of an XYZ gantry robot with programmed 1 mm movements between each burn region. The 450 nm laser was operated by using the automated engraving software with 3 mm lines programmed for each analytical repeat. The soldering iron was operated by hand and allowed to return to its set temperature after each analytical repeat and cleaned using a wet cloth between samples. All modalities were operated for 5 s for leaf material analysis.

### Rapid Evaporative Ionization Mass Spectrometry Analysis

All heating modalities were fitted with a bespoke-fitted aspirator resin head made with an SL1S three-dimensional (3D) printer (Prusa Research, Czech Republic), which gave a clearance of approximately 2 mm between the sample and opening. Approximately 2 m of poly­(tetrafluoroethylene) (PTFE) tubing with 1.5 mm internal diameter linked the aspirator head to the inlet of the Xevo G2-XS quadrupole time-of-flight (QToF) mass spectrometer (Waters, U.K.). Just prior to the inlet, the analyte-containing smoke was mixed with 2-propanol solvent^21^ containing leucine enkephalin at a concentration of 0.1 ng/μL in a stainless-steel T-piece. The 2-propanol solvent was introduced at a flow rate of 200 μL/min using an Acquity I-Class Plus binary solvent manager (Waters). The combined mixture entered the REIMS interface (Waters, U.K.), where it was heated to approximately 700 °C via collision with a Kanthal ribbon surface to remove the 2-propanol solvent prior to entry into the ion guide of the mass spectrometer. Prior to use each day, the mass spectrometer underwent calibration and detector voltage setup using the manufacturer’s recommended procedure. Mass spectra were acquired over a 50 to 1200 *m*/*z* range at a scan rate of 2 scans per second in negative ion detection mode with an instrument resolution of approximately 20,000.

### Optimization of Heating Modalities

The operating parameters of the three heating modalities were tested against healthy tomato plant leaves. For each modality, combinations of operating parameters were tested in a randomized order on a different leaf or leaf section, with six technical replicates of each completed. For the CO_2_ laser, the parameters ranged from 1.5W to 3.0W of laser power at 0.5W increments, and pulsatile operation at 1, 5, 10, 30, 50, 100, and 250 Hz. For the soldering iron, only the heating temperature in a gradient of 200 to 450 °C, in 50 °C increments, was tested. For the 450 nm laser, only the laser depth setting could be adjusted by the operating software, which was tested at 50, 75, and 100% depth. Weights of ablated material were calculated for optimized parameters by weighing a leaf section pre- and postanalysis using an analytical balance accurate to 0.1 mg.

### Infection with 

Population stocks of were maintained across their lifecycle within the School of Biological Sciences at Queen’s University Belfast. Nematodes for this project were sourced from this population and used to infect the tomato plants. A total of 12 plants were each infected with 500 nematodes (based on microscopy counts) per pot. Alongside 12 control plants, these were kept in mixed growth chambers until one leaf per plant was harvested at 14 days post infection for analysis. For REIMS analysis, only the soldering iron and 450 nm laser were used as heating modalities, with eight technical replicates of each leaf used in the resulting data analysis.

### Infection with 

A glycerol stock of (pathovar tomato DC3000) was grown on King’s B media agar overnight at 30 °C in an aerobic atmosphere. A single colony was transferred into liquid King’s B medium and grown overnight in the same conditions. Serial dilution plate counts were completed and the culture was adjusted to 10^5^ colony-forming units per mL in 10 mM magnesium chloride for infection. Mature plants were placed inside a protective chamber, and the total surface areas of all leaves were sprayed with the solution and allowed to dry. A total of 12 plants were infected, and 12 were used as a control, with plants mixed between growth chambers. Leaves were removed from the same height of each plant at 1, 2, 3, 5, 7, and 14 days post infection and analyzed using optimized heating modalities. For REIMS analysis, only the soldering iron and 450 nm laser were used as heating modalities, with eight technical replicates of each leaf performed, and all used for data analysis.

### Data Processing and Statistical Analysis

Acquired mass spectra were imported into Abstract Model Builder software (Version 1.0.1966.0, Waters, Hungary), where individual acquisition windows were manually selected and then underwent lockmass correction to 554.2615 *m*/*z*, background subtraction, and peak binning to 0.1 Da width bins. Any features that significantly correlated (Spearman’s coefficient < −0.5 or >0.5, with Bonferroni corrected *P* value <0.05) with leucine enkephalin intensity and/or run order were removed using a custom R pipeline. For parameter optimization, data was analyzed in GraphPad Prism (version 10.0.1). For statistical analysis, data was uploaded to the online MetaboAnalyst 5.0 ^22^ workflow. Data was subjected to total ion count normalization, Log10 transformation, and Pareto scaling prior to univariate analysis through Mann–Whitney *U* test with FDR multiple hypothesis correction (with a significance threshold of *p* < 0.05) and multivariate principal component analysis (PCA) and sparse partial least-squares discriminant analysis (sPLS-DA), which were validated with leave-20%-out cross-validation based on replicates across all samples. MetaboAnalyst does not support a leave-one-leaf-out cross-validation, which would be more statistically robust. Biomarker analysis was completed within the MetaboAnalyst workflow using the same preprocessing parameters. Where required, tentative metabolite annotations were assigned from the Human Metabolome Database^23^ (HMDB), with a mass accuracy threshold of <10 ppm alongside literature searches of potential matches.

### Safety Considerations

Both the CO_2_ and 450 nm lasers were categorized as Class IV instruments, and the necessary containments and safety precautions were made. The CO_2_ laser was operated within a transparent plexiglass cabinet made with 10 mm thick material, with manual firing of the laser using a foot pedal. The 450 nm laser was operated by software control inside a 3 mm green plexiglass cabinet. Both of these cabinets were sufficient to block an errant laser beam. All solvents were handled according to their material safety data sheet provided by their respective manufacturer. Plant pathogens were handled within containment level 2 facilities and according to our authorization to hold plant pathogens (PHA-420) by the Northern Ireland Department of Agriculture, Environment, and Rural Affairs.

## Results and Discussion

### Optimization of Heating Modalities

Three modalities were tested against tomato plant leaf material to optimize the heating parameters. A total of six replicates for each combination of parameters were completed. Summary figures for the best-performing parameter combinations, based on total ion count intensity (TIC) and number of features above set intensity thresholds (above 10^3^, 10^4^, 10^5^, and 10^6^, respectively), for each heating modality are given in Figure 1, with a full breakdown given in Supporting Figure S1. Across all three, relatively similar levels of TIC and feature threshold intensities were observed. For the CO_2_ laser, Figure 1a–c, a higher frequency of laser pulsatile operation was associated with both a lower TIC and a lower number of features meeting a high intensity threshold, particularly at lower laser powers. This suggests that longer laser pulses are needed in order for the heating temperature to reach a critical point for the release of metabolites for ionization, which has been observed in other biomasses using a CO_2_ laser for heating during REIMS analysis.^24^ The analysis region from the CO_2_ laser, Figure 1a, can be seen to be highly reproducible due to its integration with an automated XYZ gantry robot. For the CO_2_ laser, 3W at 5 Hz was chosen as the optimal heating parameter. Fewer parameter combinations were available for testing for the 450 nm laser, with only the laser depth setting available to change in the control software, which is used as a proxy of power, with settings of 50, 75, and 100% available. This means an approximate range of 1.5–3.0W of laser power was tested. Based on all three parameters as shown in Figure 1d–f, the 100% depth setting resulted in a considerably higher TIC and number of features above set threshold intensities, substantially at 10^5^ and 10^6^ thresholds. A higher TIC variability is, however, observed at this setting, which may be linked to the observed variability in analytical repeats, as shown in Figure 1h. Similar levels of variability were observed for soldering iron analysis which, due to its hand-held operation, is largely expected. Although variability was higher for both 450 nm laser and soldering iron heating, it is still within a relatively small window for the 450 nm laser (between ≈ (1–2) × 10^9^ TIC), while for soldering iron heating it was a 4-fold (between ≈ (2–8) × 10^9^ TIC) range. For soldering iron heating, a temperature of 400 °C was determined as optimal through a combination of the highest TIC intensity and observed variability of measurements.

**Figure 1:**
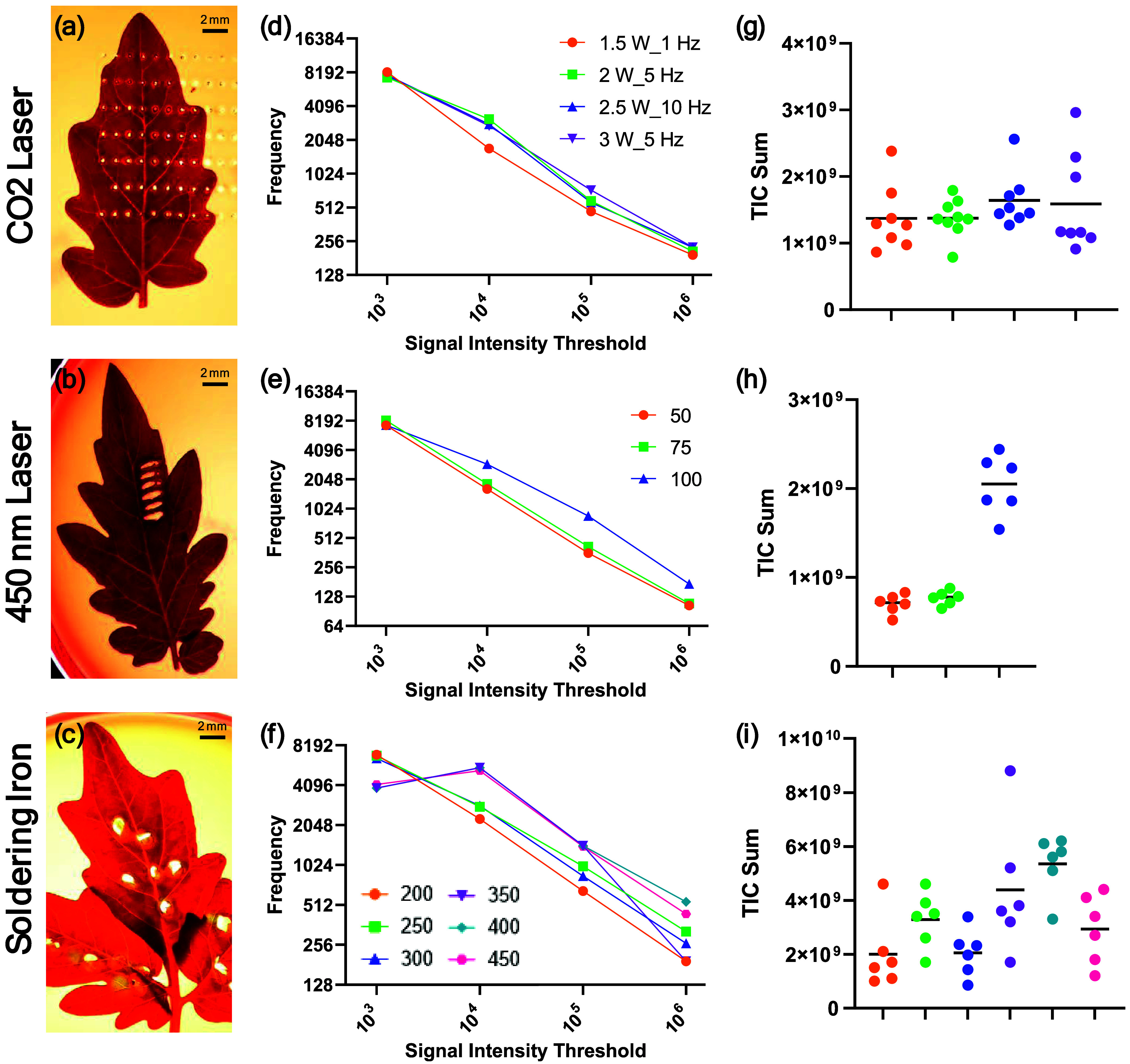
Optimization of three heating modalities for the plant leaves. Heating optimization was completed using different combinations of operating conditions for each heating modality tested. For the CO_2_ laser, a selection of the highest performing conditions is given, with all available in Supporting Figure S1. For each of the CO_2_ laser, 450 nm laser, and soldering iron respectively, panels (a–c) give a representative image of a leaf postanalysis with each modality with 2 mm scale bar; (d–f) show the number of features identified above a set threshold of intensity; and (g–i) show the total ion count intensity. The color legends are shared between panels for each heating modality.

### Comparison of Optimized Parameters

The REIMS metabolite fingerprinting data from the three optimal parameters for each heating modality were compared, Figure 2. This was to determine whether there were differences in the detected profiles among the three. The CO_2_ and 450 nm lasers showed similarities in both TIC intensity and frequencies of features above certain thresholds. The soldering iron had both a higher TIC intensity and features above all thresholds, except 10^3^, as shown in Figure 2a,b. The variability in signal intensity was, however, higher than that of both lasers, which may reflect the manual use of the soldering iron compared to the automated analysis workflows for both lasers. The weight of the ablated material for each modality is shown in Supporting Figure S2 and corresponds largely to the surface area of analysis. Similar levels of reproducibility are shown in ablated material, however, which may suggest that the higher variability in signal intensity for the soldering iron approach comes from impacts on ionization or ion fragmentation during heating. PCA modeling of the metabolite fingerprinting data, Figure 2c, shows clear separation between all three heating modalities, with the soldering iron having the largest degree of variation. When the top 1000 features, based on mean intensity across all replicates, found in the metabolite fingerprints of all three modalities are compared, Figure 2d, it can be seen that the majority of features (53.9%) are shared between the three. The remainder are either solely found in each modality’s top 1000 features based on intensity, with the 450 nm laser accounting for the highest number of these at 22.3%, or shared between two, with the greatest overlap between the CO_2_ laser and soldering iron at 18.8% of features. Based on these results, all three heating modalities appear to be effective at heating and generating an analyte-containing smoke from plant material. Although all three produce different metabolite fingerprints, the majority of features are shared. Based on visual inspection of raw and processed mass spectra in Supporting Figures S3 and S4, respectively, the main differences are not the presence/absence of features but rather their relative intensities. We do not, therefore, believe that different ionization mechanisms are at work, but rather that each modality differs in the intensity of ions generated. This is further supported by the spread of PC loadings across the mass-to-charge range shown in Supporting Figure S5 from Figure 2c. The automated laser modalities have lower variation compared to the hand-held soldering iron but come with associated laser hazards that require additional considerations for containment. For subsequent analysis of leaves from infected plants, it was decided to continue only with the 450 nm laser and soldering iron heating modalities. This is because they are the lowest cost of the three, and smaller and more moveable than the CO_2_ laser, which would potentially make them easier to adapt for *in situ* analysis.

**Figure 2:**
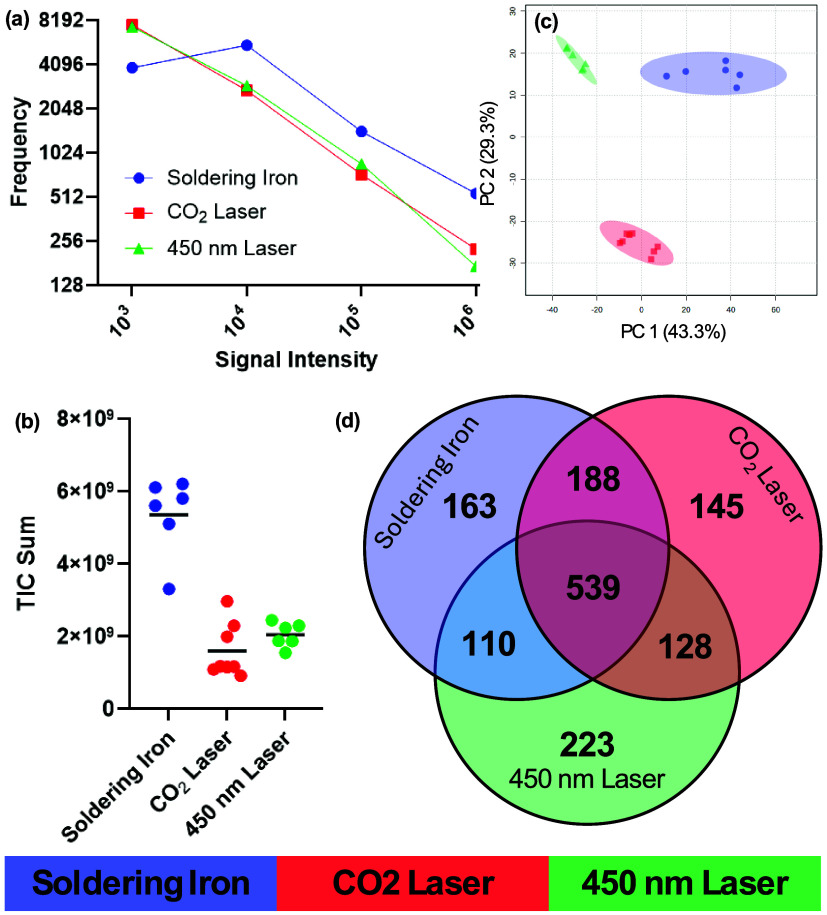
Comparison of REIMS spectral fingerprints between optimized parameters for all three modalities. Panel (a) shows the number of features above set intensity thresholds; panel (b) compares total ion count intensities; panel (c) gives a 2D scores plot from principal component analysis of the three modalities; and panel (d) shows the number of features solely found within the top 1000 features by intensity for each modality, or shared between modalities.

### Biomarkers for Parasitic Infection

Plant parasitic nematodes infect the roots of plants and are difficult to diagnose due to a typical requirement to identify root nodules below the soil surface. In this work, we infected mature tomato plants with a moderate dose (500) of worms per plant. These were then incubated for 14 days to mimic an early infection time point.^25^ A leaf from each plant was removed and analyzed for each of the 450 nm laser and soldering iron heating modalities using optimized conditions. To understand the impact on the overall plant metabolome as a result of infection, we first undertook multivariate analysis of our data using sparse partial least-squares discriminant analysis (sPLS-DA). This is similar to PLS-DA analysis but uses a reduced set of features selected based on their classification performance across multiple rounds of cross-validation to reduce the risk of overfitting models.^26^ Through this analysis, the 450 nm laser has superior performance (0.6% error rate) against the soldering iron (14.0% error rate) with a clear ability to significantly separate between control and infected plant material, as shown in Figure 3a,b. Although valuable, a multivariate approach using a large number of features for classification would likely require a mass spectrometer with untargeted mass analyzing ability and reduce the potential for in-field or *in situ* analysis. Further analysis explored the ability of univariate features through an area under the receiver operating characteristic (ROC) curve (AUC) approach. Using a cutoff of greater than 0.8 as a diagnostically useful threshold, the 450 nm laser again showed a much greater diagnostic potential than the soldering iron, with 491 features above this threshold, and of these, 112 above 0.9 and 18 above 0.95, as shown in Figure 3c,d. The soldering iron showed no features above the 0.8 threshold, as shown in Figure 3d,f. There is total overlap between the 20 features in component 1 of the sPLS-DA model that drive the significant separation between infected and healthy plant material and the highest ranking AUC features, as shown in Supporting Figure S6. This is further supported by univariate Volcano plots comparing features significantly higher in control or infected plants for both modalities, shown in Supporting Figure S7.

**Figure 3:**
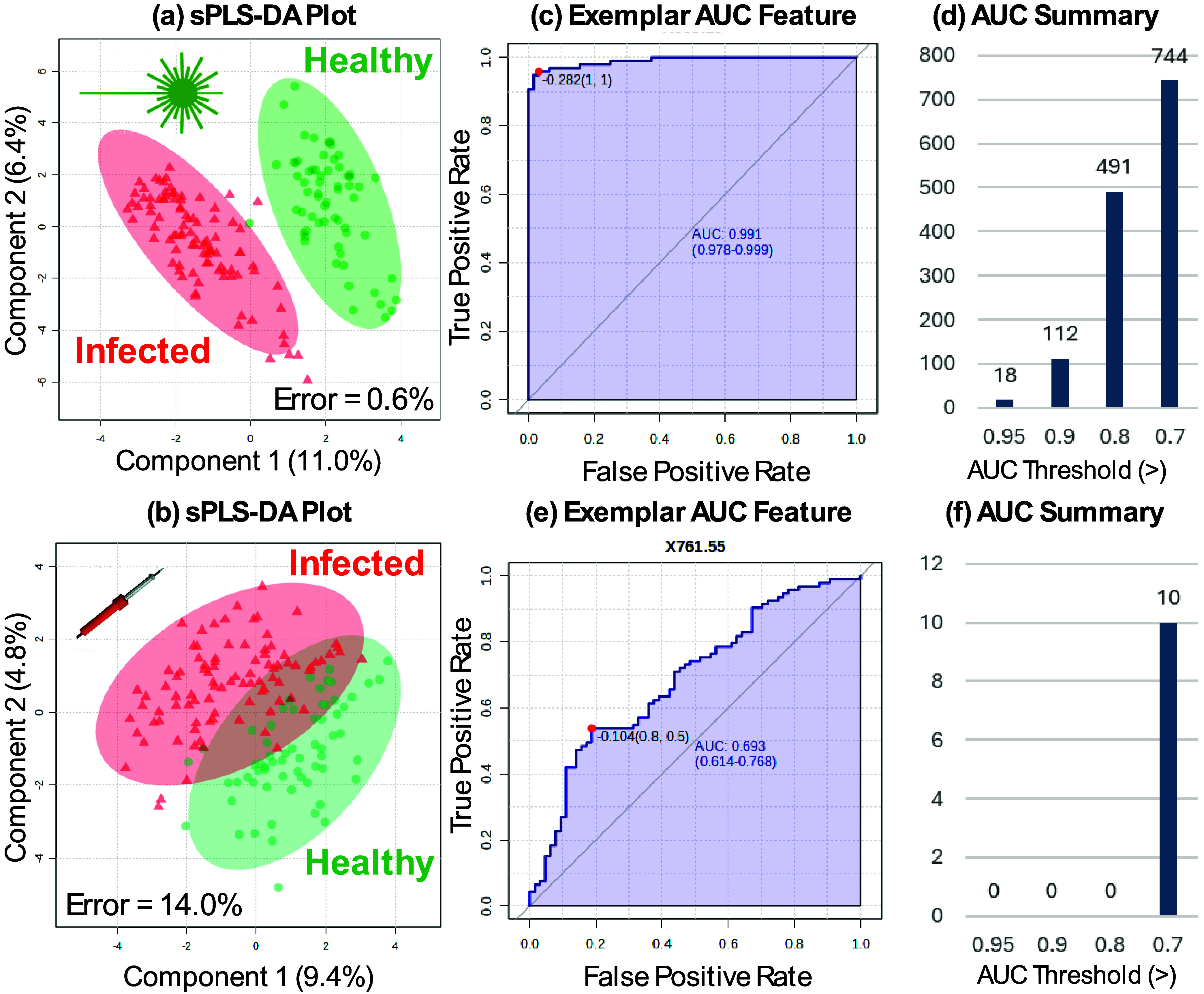
Effectiveness of REIMS using either a 450 nm laser or a soldering iron to identify biomarkers indicative of infection. Multivariate using sparse partial least-squares discriminant analysis (sPLS-DA) is compared between (a) 450 nm laser and (b) soldering iron, with cross-validation error rates given for each. An exemplar area under the receiver operating characteristic curve (AUC) is given for (c) 450 nm laser and (d) a summary of the total number above three thresholds; and for (e) soldering iron with representative AUC and (f) total summary.

### Biomarkers for Bacterial Infection

Bacterial pathogens typically impact their hosts at an earlier time point following infection. This is largely based on their ability to grow at a faster rate. Unlike , infection by occurs through the leaf material, where it penetrates and forms an aqueous apoplast in the intracellular tissues of leaves and stems.^27^ We used a spray inoculation technique to infect mature tomato plants and incubated them for 14 days alongside uninfected control samples, with analysis at 1, 2, 3, 5, 7, and 14 days post infection (dpi). This would allow the detection of very early-stage biomarkers of infection and to determine whether these persist over a time course of infection. From multivariate sPLS-DA modeling, no significant separation between infected and uninfected plants was observed at any time point post infection, as shown in Figure 4a–c. The cross-validation error rate varied between 7.8 and 12.9%, which suggests that models are robust but lack a very high level of accuracy. As with infections, we also completed univariate analysis through a biomarker AUC approach, using a value above 0.8 as a clinically useful threshold. A large number of biomarkers (162) were observed above this threshold for at least one time point, with a total of 54 observed across all six time points, as shown in Figure 4d. In line with performance for infection, the soldering iron modality performed poorly for . sPLS-DA modeling shows high error rates above 25% at all time points and with no individual feature showing an AUC value above 0.7, as shown in Supporting Figure S8. Univariate Volcano plots comparing features significantly higher in control or infected plants for both modalities are shown in Supporting Figure S9 and further support that the 450 nm laser identifies a higher number of features significantly higher in infected plants than the soldering iron modality is capable of (Figure 4).

**Figure 4:**
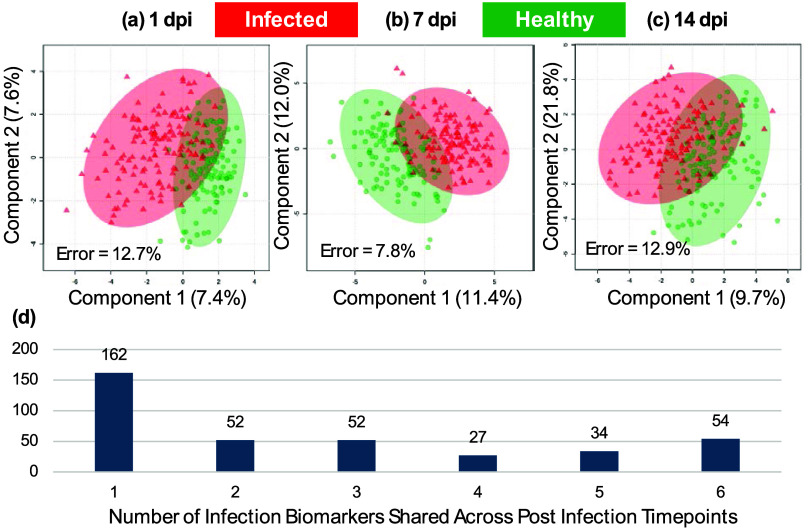
Effectiveness of 450 nm laser REIMS analysis to differentiate between plants infected by and healthy plants. Six time points were analyzed with sPLS-DA models and associated error rates given for (a) 1 day post infection (dpi); (b) 7 dpi; and (c) 14 dpi. The number of univariate features that exceeded a threshold of >0.8 are given in panel (d) as a total against the number of post infection time points they are identified in.

### Biochemical Underpinning of Diagnostic Markers

In this work, we have compared the REIMS metabolic fingerprint collected from the same host species of tomato plant when infected with two different pathogens. This allows a comparison between the diagnostically valuable biomarkers for each pathogen to determine whether we detect pathogen-specific biomarkers or just universal biomarkers of biotic stress and infection. Comparing the features identified for at the 14 dpi time point and infection across all six post infection time points, as shown in Figure 5, the largest number (452) were unique to infection. For , 15 features were uniquely identified for this pathogen, and 39 were shared across both pathogens. This suggests that through REIMS analysis, we are able to identify pathogen-specific biomarkers at diagnostically useful time points following infection. To provide further weight to the evidence supporting this, we explored tentative annotations of identified features through accurate mass matches against the HMDB. Although it is not a plant-specific database, it is the most extensive metabolite database available to the community and contains a large number of diet-associated, and therefore, plant-associated metabolites. Looking first at the features unique to the altered metabolome of tomato plants following infection, we tentatively identified the top ten ranked features based on AUC values (Supporting Table S1). Within these we tentatively annotated an adduct of 2-furanylmethyl butanoate which is a fatty acid ester and interestingly, field trials have shown that the application of fatty acid esters reduces the productivity loss associated with infection.^28^ We also tentatively identified an adduct of succinyldisalicylic acid, which has been previously associated with root knot nematodes in tomato plants.^29^ In addition to these, several complex lipids were tentatively identified, including DG(35:6), CerP­(d44:2), PG(34:1), and DG(42:2), which have all been implicated in plant response to pathogens.^30,31^ A smaller number of features were identified uniquely as indicative of infection in tomato plants, and of these 15, we were able to tentatively annotate only two (Supporting Table S2). One of these was determined as an adduct of TG(64:2), and triacylglycerols have been previously shown to accumulate at the site of infection in plants.^32^ The other feature was tentatively identified as an adduct of lysoPC(28:0), which is an intermediary in plant signaling pathways and has previously been shown to be involved in plant response to infection, albeit in the model .^33^ Albeit tentative, the biochemical annotations of diagnostic features align with previously reported findings associated with plant infections by the same pathogens. This supports the finding that REIMS analysis is capable of detecting metabolites directly associated with infection and that classification models/features are not based on analytical noise. However, a large number of features were not identifiable, and this highlights the need for future work to develop a plant-specific database of metabolites detectable through REIMS to support the community’s use of this novel tool.

**Figure 5:**
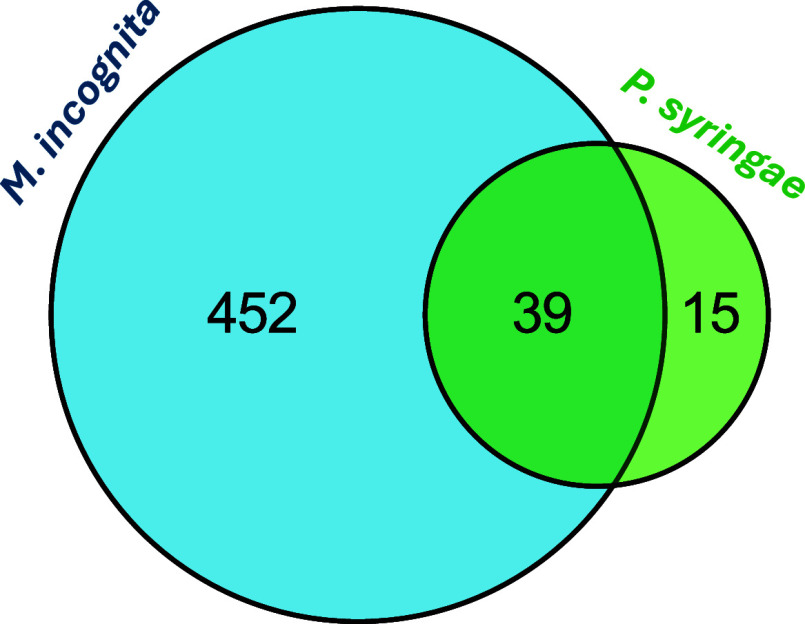
Venn diagram showing the number of unique and shared diagnostic features identified for and pathogen infections.

## Conclusions

Ambient ionization techniques hold considerable promise in expanding the scope of applications in which mass spectrometry can be of benefit. REIMS offers a particularly flexible technique in that the method of heating can be easily adapted to suit the type of sample under analysis. Here, we have shown that two very low-cost methods of heating can be used for the analysis of plant material. The use of a soldering iron did not, however, perform well in identifying markers of infection, and this may be due to the hand-held nature of the tool and the inherent variability associated with it. Lasers have been shown to be capable of conducting REIMS analysis of a broad range of samples but have typically been very high cost. We used a low-cost 450 nm laser that is typically used for the etching of wood and other materials to successfully analyze plant material and identify pathogen-specific diagnostic biomarkers. Importantly, our models relied on individual univariate AUC models and, thus, would not require a mass spectrometer capable of untargeted analysis. This offers the potential that REIMS could be deployed with a low-cost 450 nm laser and a single or triple quadrupole instrument that is typically less expensive and more robust than profiling instruments. Although this would be beneficial for a diagnostic platform, our work not only shows the potential of low-cost lasers for broader REIMS analysis but also the potential of REIMS for the analysis of plant material *in situ*. This could offer a number of potential benefits associated with speed, resources, and reducing sources of potential bias associated with storage and processing when compared to conventional mass spectrometry techniques paired with preionization chromatographic separation.

## Supplementary Material



## Notes

Includes representative mass spectra from analysis and additional statistical analysis of infection models (PDF)
