# Supplementary material for: Low-Cost Heating Modalities Allow the Detection of Biomarkers for Plant Infection Using Rapid Evaporative Ionization Mass Spectrometry (REIMS) That Are Pathogen Specific
Source: Anal Chem. 2025 Jun 27;97(27):14230–8. doi: 10.1021/acs.analchem.5c00226 (PMC12268832; doi:10.1021/acs.analchem.5c00226)
Supplement: Supplementary file 1 [file ac5c00226_si_001.pdf]

Supporting Information for:

Low-cost heating modalities allow the detection of biomarkers for plant infection using rapid evaporative ionization mass spectrometry (REIMS) that are pathogen specific

Alice Flint[1], Ryan Weir[1], Luis AJ Mur[2], Simon JS Cameron[1*]

[1] School of Biological Sciences, Institute for Global Food Security, Queen's University Belfast, Belfast, BT9 5DL, United Kingdom; [2] Department of Life Sciences, Aberystwyth University, Aberystwyth, SY23 3FL, United Kingdom.

**Supplementary Figure S1.** Full range of combinations of operating parameters for CO2 laser analysis during optimization experiments with sum of total ion count (TIC) given in top row and number of features above set intensity thresholds given in bottom row.	**2**
**Supplementary Figure S2.** Comparison of weight of ablated leaf material using each heating modality based on their optimized parameters. Six replicates were taken with weights of leaf taken pre- and post-analysis on an analytical balance accurate to 0.1 mg. Statistical analysis conducted using one-way ANOVA test (after normal distribution confirmed with Shapiro-Wilk ($p > 0.05$) test). Comparison between heating modalities conducted with *post hoc* Holm-Šídák's multiple comparisons test with p values annotated on graph.	**3**
**Supplementary Figure S3.** Representative negative ion detection raw mass spectra from optimized conditions of three heating modalities tested taken from MassLynx. The regions of (a) 50 to 550 *m/z* and (b) 560 to 1200 *m/z* are shown for clarity.	**4**
**Supplementary Figure S4.** Representative negative ion detection mass spectra from optimized conditions of three heating modalities tested after peak picking, lock mass correction, and background subtraction using MassLynx. The regions of (a) 50 to 550 *m/z* and (b) 560 to 1200 *m/z* are shown for clarity.	**5**
**Supplementary Figure S5.** Principal component loadings used to construct Figure 2c for (a) principal component 1 which explained 43.3% of the variation and (b) principal component 2 which explained 29.3% of the variation. To avoid figure saturation, only bins which had a contribution greater than 0.02 or less than -0.02 were plotted for each component.	**6**
**Supplementary Figure S6.** Top 20 features identified as important in sparse partial least square discriminant analysis (sPLS-DA) models for *Meloidogyne incognita* infection alongside their corresponding area under the receiver operating characteristic curve (AUC) value.	**7**
**Supplementary Figure S7.** Volcano plots for *Meloidogyne incognita* infections comparing significantly different features (FDR-corrected p value < 0.05) with a minimum Log_2 fold-change of 1 for (a) 450 nm laser and (b) soldering iron. Significant quadrants are annotated based on whether they are higher in control (blue) or infected (red) plants. The top 20 features are annotated to avoid figure saturation.	**8**
**Supplementary Figure S8.** Effectiveness of soldering iron REIMS analysis to differentiate between plants infected by *P. syringae* and healthy plants. Six timepoints were analyzed with sPLS-DA models and associated error rates given for (a) 1 day post infection (dpi); (b) 7 dpi; and (c) 14 dpi.	**9**
**Supplementary Figure S9.** Volcano plots for *Pseudomonas syringae* infections (1 day post infection) comparing significantly different features (FDR-corrected p value < 0.05) with a minimum Log_2 fold-change of 1 for (a) 450 nm laser and (b) soldering iron. Significant quadrants are annotated based on whether they are higher in control (blue) or infected (red) plants. The top 20 features are annotated to avoid figure saturation.	**10**
**Supplementary Table S1.** Tentative annotations of the top 10 REIMS features/bins based on ROC-AUC values for infection with *M. incognita* using the 450 nm laser modality using matches against the Human Metabolome Database (HMDB). Where there are multiple matches, the one taken was based on literature searches that associate them with previously identified associations with plant response to pathogen infection.	**11**
**Supplementary Table S2.** Tentative annotations of the top 10 REIMS features/bins based on ROC-AUC values for infection with *P. syringae* using the 450 nm laser modality using matches against the Human Metabolome Database (HMDB). Where there are multiple matches, the one taken was based on literature searches that associate them with previously identified associations with plant response to pathogen infection.	**12**

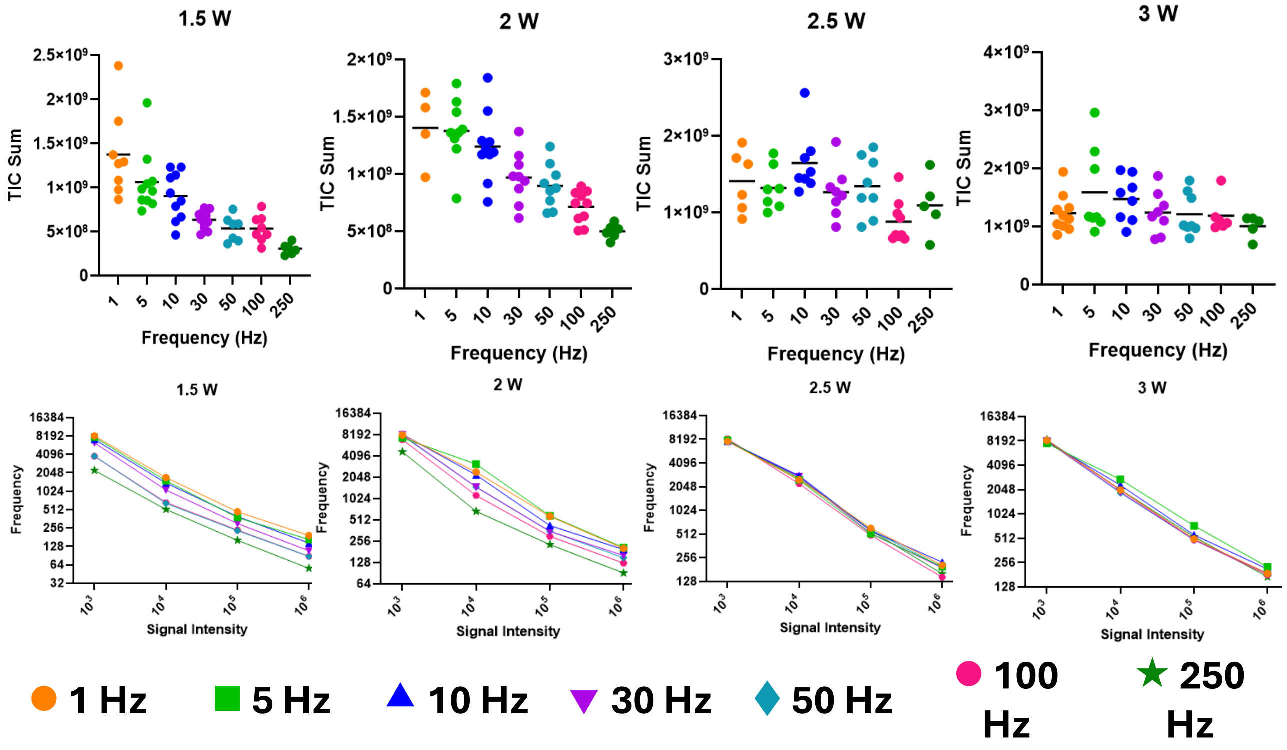

Supplementary Figure S1. Full range of combinations of operating parameters for CO2 laser analysis during optimization experiments with sum of total ion count (TIC) given in top row and number of features above set intensity thresholds given in bottom row.

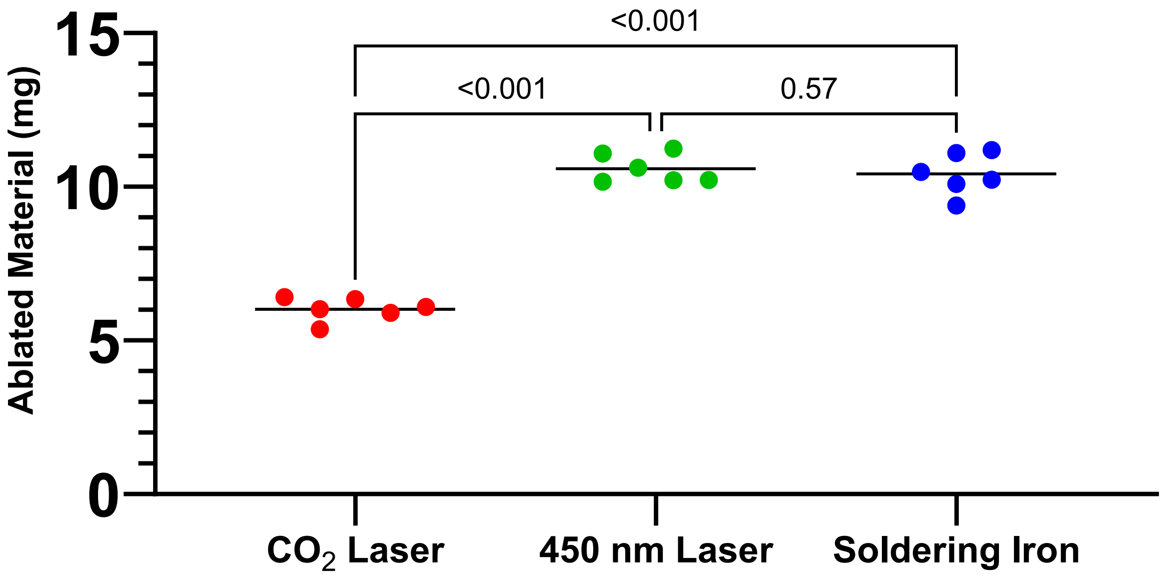

Supplementary Figure S2. Comparison of weight of ablated leaf material using each heating modality based on their optimized parameters. Six replicates were taken with weights of leaf taken pre- and post-analysis on an analytical balance accurate to 0.1 mg. Statistical analysis conducted using one-way ANOVA test (after normal distribution confirmed with Shapiro-Wilk ($p > 0.05$) test). Comparison between heating modalities conducted with *post hoc* Holm-Šídák's multiple comparisons test with *p* values annotated on graph.

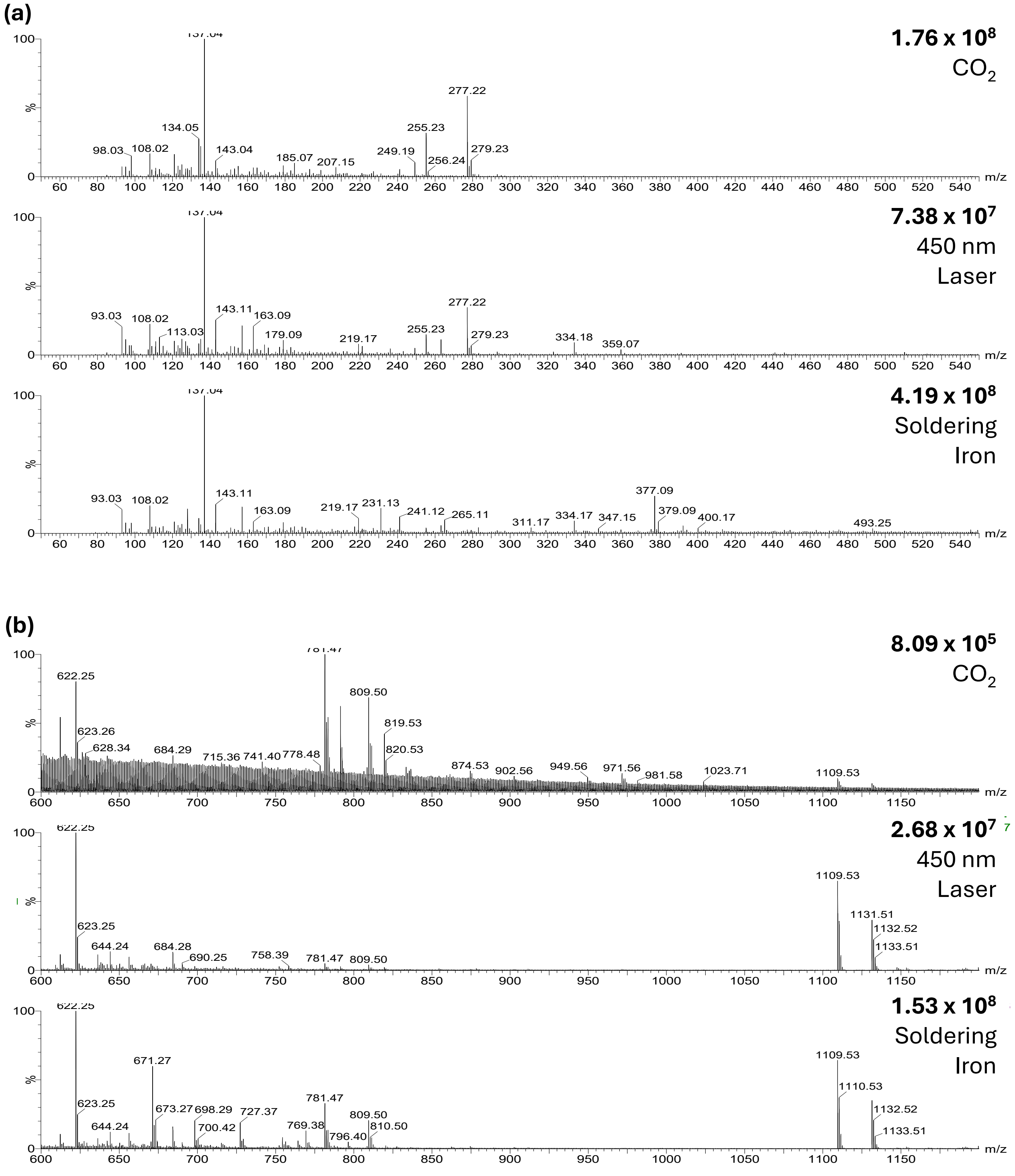

Supplementary Figure S3. Representative negative ion detection raw mass spectra from optimized conditions of three heating modalities tested taken from MassLynx. The regions of (a) 50 to 550 *m/z* and (b) 560 to 1200 *m/z* are shown for clarity.

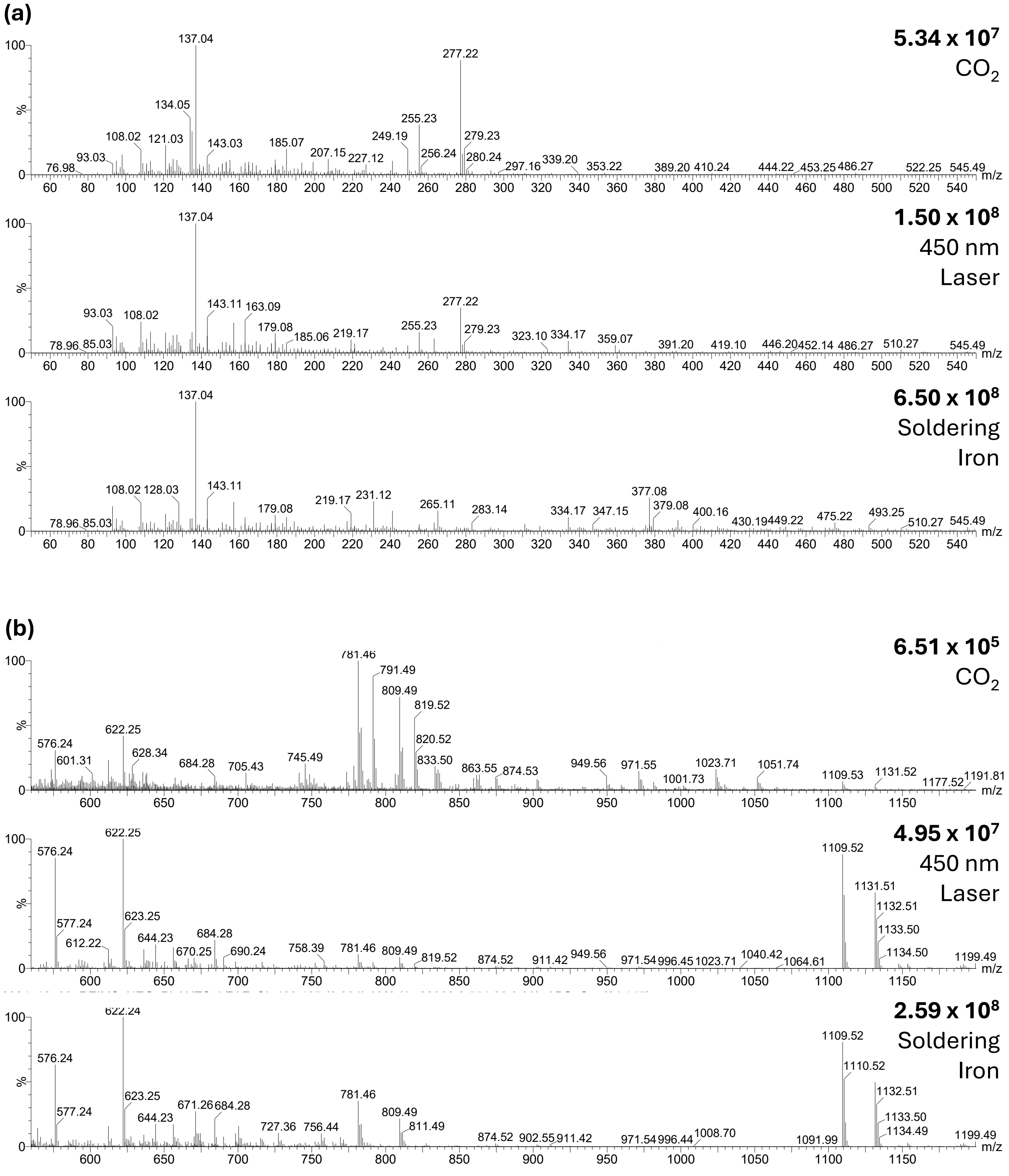

Supplementary Figure S4. Representative negative ion detection mass spectra from optimized conditions of three heating modalities tested after peak picking, lock mass correction, and background subtraction using MassLynx. The regions of (a) 50 to 550 *m/z* and (b) 560 to 1200 *m/z* are shown for clarity.

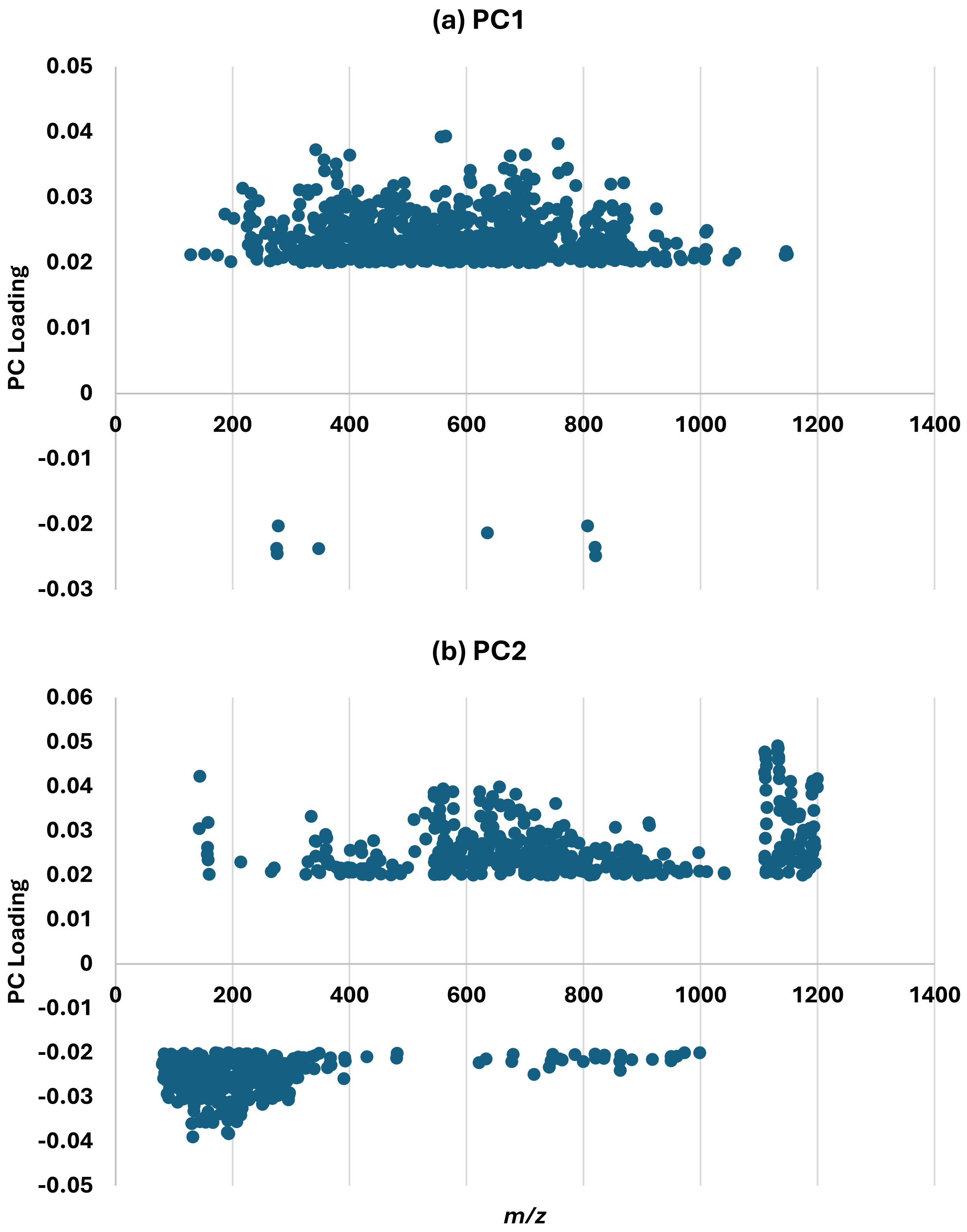

Supplementary Figure S5. Principal component loadings used to construct Figure 2c for (a) principal component 1 which explained 43.3% of the variation and (b) principal component 2 which explained 29.3% of the variation. To avoid figure saturation, only bins which had a contribution greater than 0.02 or less than -0.02 were plotted for each component.

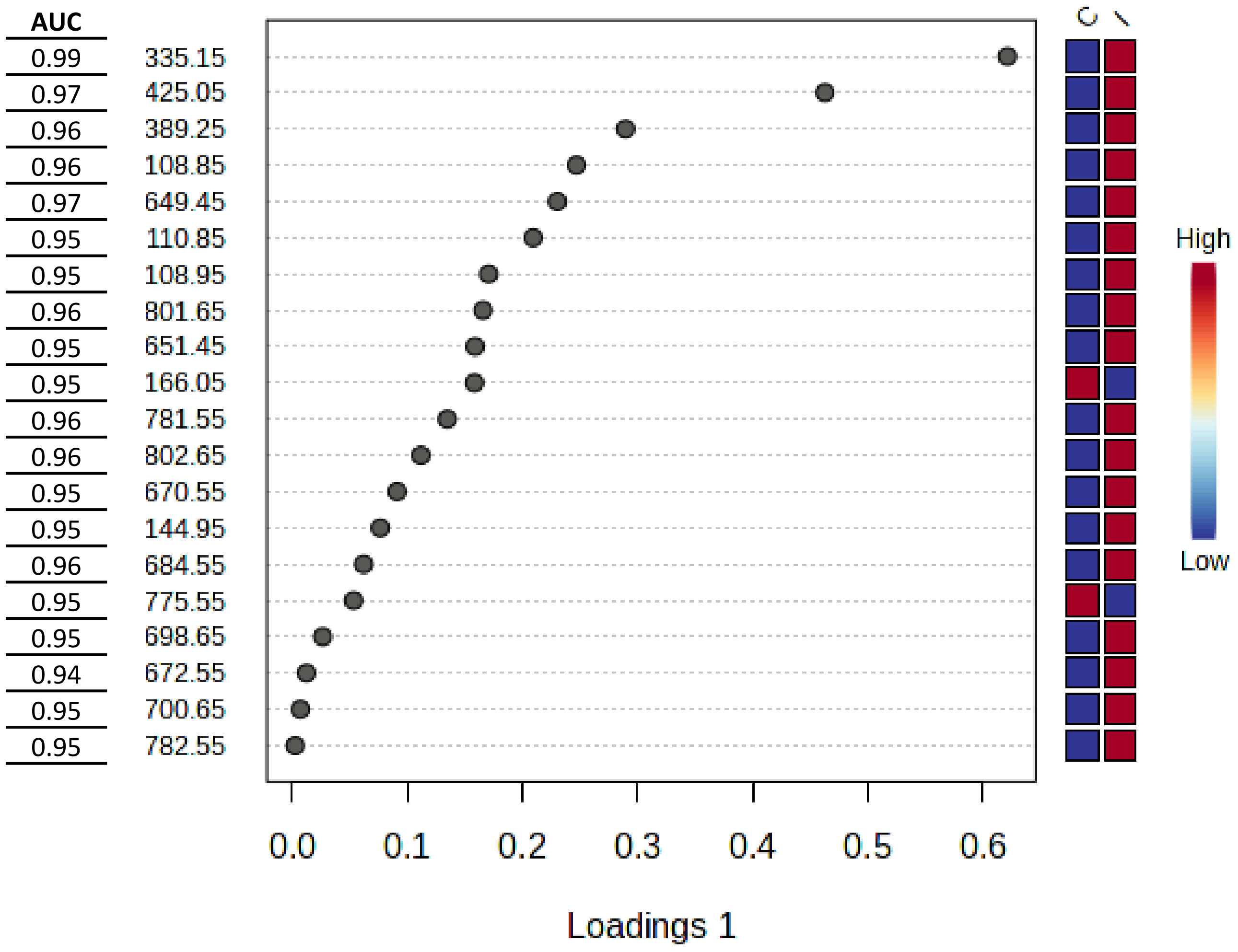

Supplementary Figure S6. Top 20 features identified as important in sparse partial least square discriminant analysis (sPLS-DA) models for *Meloidogyne incognita* infection alongside their corresponding area under the receiver operating characteristic curve (AUC) value.

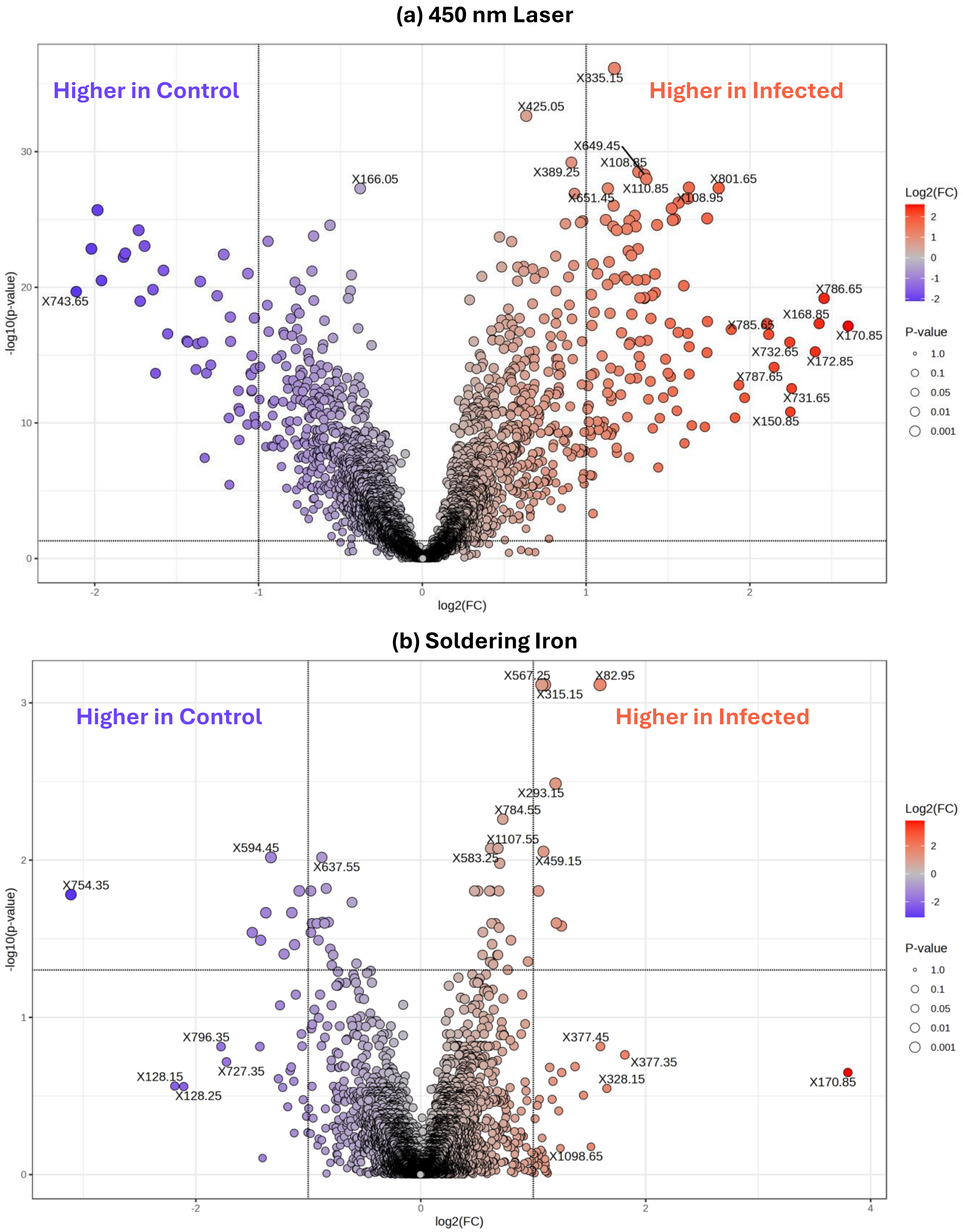

Supplementary Figure S7. Volcano plots for *Meloidogyne incognita* infections comparing significantly different features (FDR-corrected *p* value < 0.05) with a minimum Log_2 fold-change of 1 for (a) 450 nm laser and (b) soldering iron. Significant quadrants are annotated based on whether they are higher in control (blue) or infected (red) plants. The top 20 features are annotated to avoid figure saturation.

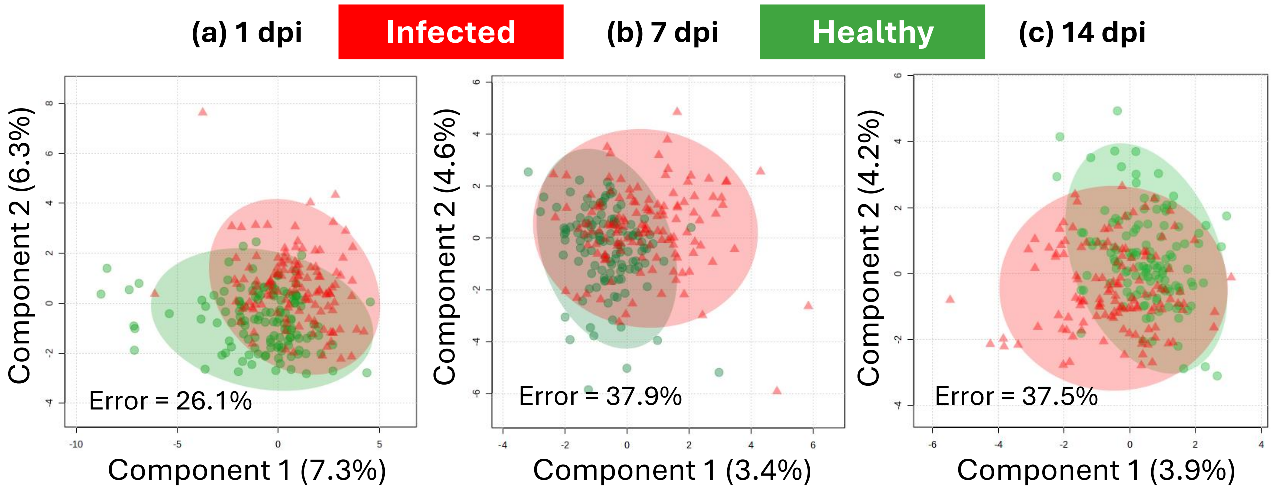

Supplementary Figure S8. Effectiveness of soldering iron REIMS analysis to differentiate between plants infected by *P. syringae* and healthy plants. Six timepoints were analyzed with sPLS-DA models and associated error rates given for (a) 1 day post infection (dpi); (b) 7 dpi; and (c) 14 dpi.

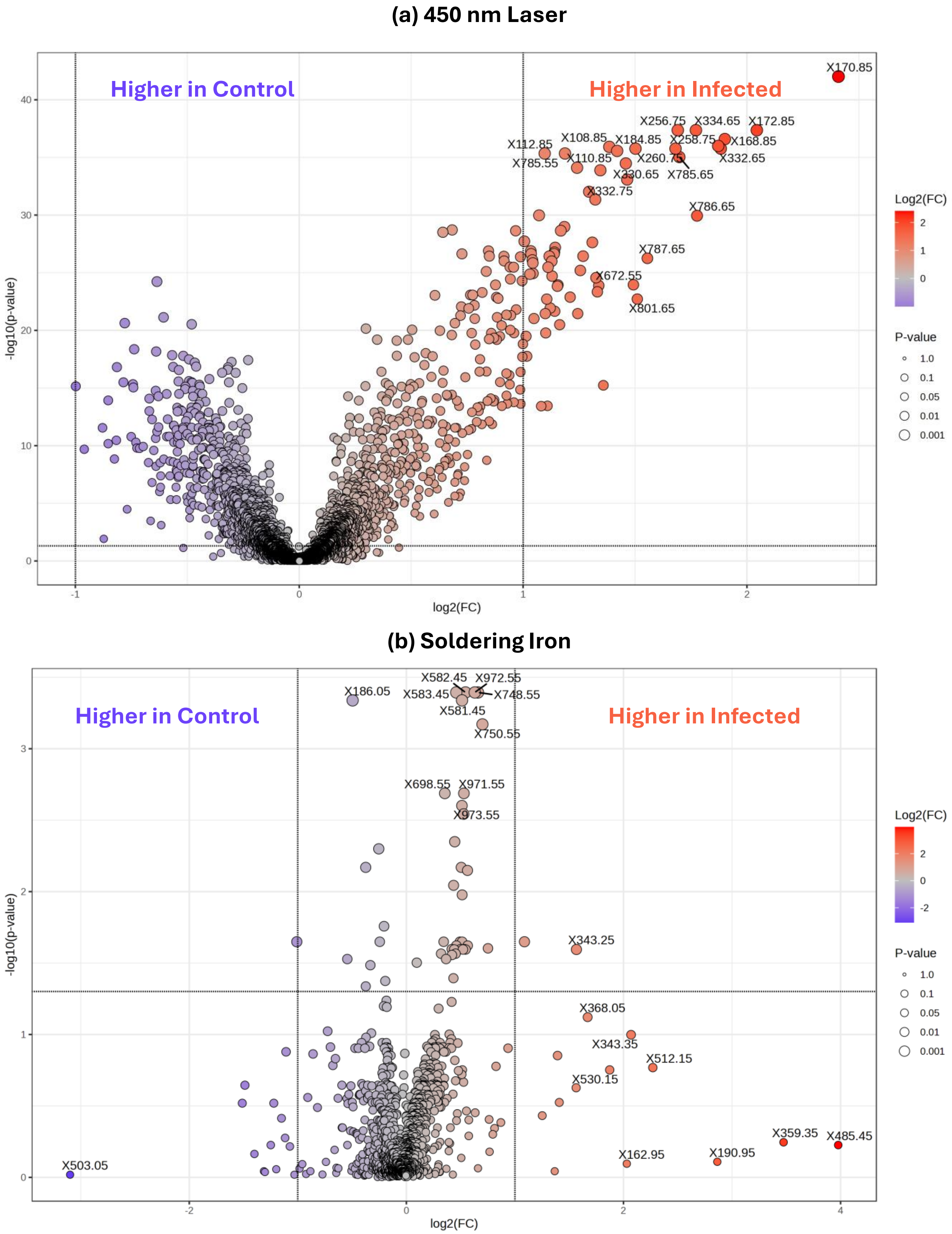

Supplementary Figure S9. Volcano plots for *Pseudomonas syringae* infections (1 day post infection) comparing significantly different features (FDR-corrected p value < 0.05) with a minimum Log_2 fold-change of 1 for (a) 450 nm laser and (b) soldering iron. Significant quadrants are annotated based on whether they are higher in control (blue) or infected (red) plants. The top 20 features are annotated to avoid figure saturation.

Supplementary Table S1. Tentative annotations of the top 10 REIMS features/bins based on ROC-AUC values for infection with *M. incognita* using the 450 nm laser modality using matches against the Human Metabolome Database (HMDB). Where there are multiple matches, the one taken was based on literature searches that associate them with previously identified associations with plant response to pathogen infection.

Query m/z	*Match*	*ppm*	*Name*	*Formula*	*Adduct*	*Single Match*
335.15	335.1500	0	2-Furanylmethyl butanoate	$C_9H_{12}O_3$	2M-H	No
649.45	649.4480	3	DG(35:6)	$C_{38}H_{62}O_7$	M+F	No
425.05	425.0491	2	Succinyldisalicylic acid	$C_{18}H_{14}O_8$	M-H+HCOONa	No
781.55	871.5501	0	SM(d38:5)	$C_{43}H_{79}N_2O_8P$	M-H	No
801.65	No Match					
389.25	780.5153	1	PG(34:1)	$C_{40}H_{77}O_{12}P$	M-2H	No
783.55	783.5508	1	DG(42:2)	$C_{45}H_{84}O_5$	M+Br	No
684.55	No Match					
108.85	No Match					
802.65	802.6490	1	CerP(d44:2)	$C_{46}H_{90}NO_6P$	M+F	Yes

Supplementary Table S2. Tentative annotations of the top 10 REIMS features/bins based on ROC-AUC values for infection with *P. syringae* using the 450 nm laser modality using matches against the Human Metabolome Database (HMDB). Where there are multiple matches, the one taken was based on literature searches that associate them with previously identified associations with plant response to pathogen infection.

Query m/z	*Match*	*ppm*	*Name*	*Formula*	*Adduct*	*Single Match*
334.65	No Match					
256.75	No Match					
330.65	No Match					
184.85	No Match					
260.75	No Match					
332.65	No Match					
182.85	No Match					
186.85	No Match					
336.65	336.6515	4	TG(64:2)	$C_{67}H_{128}O_5$	M-3H	Yes
330.75	330.7529	9	LysoPC(28:0)	$C_{36}H_{74}NO_7P$	M-2H	Yes
